# Clinical screening of Nocardia in sputum smears based on neural networks

**DOI:** 10.3389/fcimb.2023.1270289

**Published:** 2023-11-29

**Authors:** Hong Sun, Xuanmeng Xie, Yaqi Wang, Juan Wang, Tongyang Deng

**Affiliations:** ^1^ Department of Laboratory Medicine, Tongde Hospital of Zhejiang Province, Hangzhou, China; ^2^ Effect, Jianying, Intelligent Creation Lab, Bytedance Inc., Hangzhou, China; ^3^ College of Media Engineering, Communication University of Zhejiang, Hangzhou, China

**Keywords:** Nocardia, Nocardia screening, neural network, sputum specimen, Nocardia infection, nocardiosis

## Abstract

**Objective:**

Nocardia is clinically rare but highly pathogenic in clinical practice. Due to the lack of Nocardia screening methods, Nocardia is often missed in diagnosis, leading to worsening the condition. Therefore, this paper proposes a Nocardia screening method based on neural networks, aiming at quick Nocardia detection in sputum specimens with low costs and thereby reducing the missed diagnosis rate.

**Methods:**

Firstly, sputum specimens were collected from patients who were infected with Nocardia, and a part of the specimens were mixed with new sputum specimens from patients without Nocardia infection to enhance the data diversity. Secondly, the specimens were converted into smears with Gram staining. Images were captured under a microscope and subsequently annotated by experts, creating two datasets. Thirdly, each dataset was divided into three subsets: the training set, the validation set and the test set. The training and validation sets were used for training networks, while the test set was used for evaluating the effeteness of the trained networks. Finally, a neural network model was trained on this dataset, with an image of Gram-stained sputum smear as input, this model determines the presence and locations of Nocardia instances within the image.

**Results:**

After training, the detection network was evaluated on two datasets, resulting in classification accuracies of 97.3% and 98.3%, respectively. This network can identify Nocardia instances in about 24 milliseconds per image on a personal computer. The detection metrics of mAP50 on both datasets were 0.780 and 0.841, respectively.

**Conclusion:**

The Nocardia screening method can accurately and efficiently determine whether Nocardia exists in the images of Gram-stained sputum smears. Additionally, it can precisely locate the Nocardia instances, assisting doctors in confirming the presence of Nocardia.

## 1 Introduction

The Nocardia genus is a kind of aerobic, Gram-positive, weakly acid-fast, branching filamentous bacteria (Lerner, 1996; Fatahi-Bafghi, 2018). In the past decades, our understanding of the pathogenicity of Nocardia is continually deepening. In an early stage, Nocardia was believed to only infect immunocompromised patients (Paige and Spelman, 2019; Zia et al., 2019; Traxler et al., 2022). However, as research progressed, it has been discovered that Nocardia can also infect immunocompetent individuals (Fujita et al., 2016; Abe et al., 2021; Margalit et al., 2021). Nocardia infections can arise on multiple organs, including the skin (Akasaka et al., 2011; Chen et al., 2020), lungs (Abe et al., 2021; Li et al., 2022; Chen and Hu, 2023), brain (Song et al., 2021), etc. Among them, the lungs have the highest infection rate (Margalit et al., 2020; Yetmar et al., 2023), accounting for approximately 50-70% of Nocardia infections (Ambrosioni et al., 2010). This can lead to pneumonia, lung abscesses, bronchiectasis, chronic obstructive pulmonary diseases, etc. (Saubolle and Sussland, 2003) More critically, when Nocardia spreads into the bloodstream, it can cause brain infections or even death (Filice, 2001; Song et al., 2021). There is no specific characteristic in radiology of Nocardial pulmonary diseases. It can present as pulmonary nodules, consolidations, cavitary masses, pleural effusions, etc., making it difficult to be distinguished from other infections (Traxler et al., 2022).

Nocardia infection is not commonly encountered in clinical practice. Over a span of six years, from 2001 to 2006, a large teaching hospital in Miami recorded the incidence of Nocardia cases. Among the 25 reported cases, 21 involved pulmonary infections, with nine cases detected from sputum (Castro and Espinoza, 2007). On average, less than four cases were identified annually. Ercibengoa et al. (Ercibengoa et al., 2020) conducted a multicenter analysis of Nocardia pneumonia in Spain, specifically studying 55 cases from five hospitals between 2010 and 2016. The average number of infections per hospital per year was less than two.

**Figure 1:**
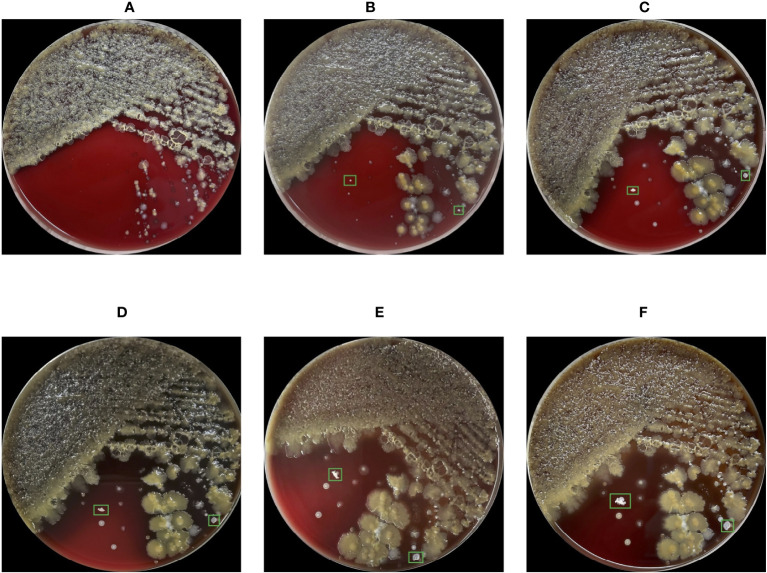
Images of the blood agar plate captured from Day 1 to Day 6 **(A-F)** during bacterial culture. Due to the low growth rate, Nocardia colonies were indistinguishable in the first 3 days, leading to missed misdiagnosis. The cultivation conditions for this bacterial culture include aerobic conditions, 35 degrees Celsius, and a 5% concentration of carbon dioxide.

The gold standard for diagnosing Nocardial pulmonary disease is bacterial culture (Jiao et al., 2021). However, Nocardia has a slow growth rate in culturing, most cultures become positive in 2-7 days, but the duration must be extended to 2-3 weeks due to slow-growing species (Rouzaud et al., 2018). In **Figure 1**, the conditions of the bacterial culture from Day 1 to Day 6 are demonstrated, and the Nocardia colonies are marked with bounding boxes. Note that the proposed method does not include the step of bacterial culturing and **Figure 1** is provided merely to show Nocardia’s low growth rate. Because of the slow growth rate, Nocardia infections are difficult to be discovered in an early stage. Current laboratory diagnostic methods for Nocardia include Matrix-Assisted Laser Desorption Ionization-Time Of Flight (MALDI-TOF) (Carrasco et al., 2016), real-time Polymerase Chain Reaction (PCR) (Wang et al., 2023b), Next-Generation Sequencing (NGS) (Saubolle and Sussland, 2003), etc. However, these methods are costly and require a high level of skill from the operator, making them unsuitable for large-scale screening.

One of the most commonly used method for Nocardia screening is manual identification based on the morphology in Gram-stained sputum smears under a microscope (Brown-Elliott et al., 2006). However, the manual identification method suffers from low efficiency and unreliability. Additionally, laboratory technicians are usually unfamiliar with Nocardia due to its rarity, resulting in missed diagnoses (Mehta and Shamoo, 2020).

In recent years, deep neural networks have been widely used in various fields, including medical engineering (Anwar et al., 2018; Boveiri et al., 2020; Kulkarni et al., 2021; Abdou, 2022; Sarvamangala and Kulkarni, 2022). They have been proven to have the advantages of reliability, efficiency and cost-effectiveness compared to traditional methods. Specifically, in medical engineering, they have been adopted for blood cell detection (Liang et al., 2018; Acevedo et al., 2019), mycobacterium tuberculosis identification (Xiong et al., 2018; Kuok et al., 2019), and many other medical applications (Rahman et al., 2020; Malhotra et al., 2022; Rho et al., 2022). However, neural networks have never been adopted for Nocardia detection, which poses new challenges: 1) the irregular morphology of Nocardia presents high diversity, making it difficult for neural networks to identify; 2) Nocardia infection is not commonly encountered in medical practice, making it difficult to collect sufficient data for network training; 3) the sputum specimens contain various cocci, bacilli, fungi, white blood cells, epithelial cells, etc., making it difficult to identify Nocardia instances. In the next section, we will illustrate how to address these challenges and demonstrate the procedures of the neural network-based Nocardia screening method.

## 2 Materials and methods

**Figure 2:**
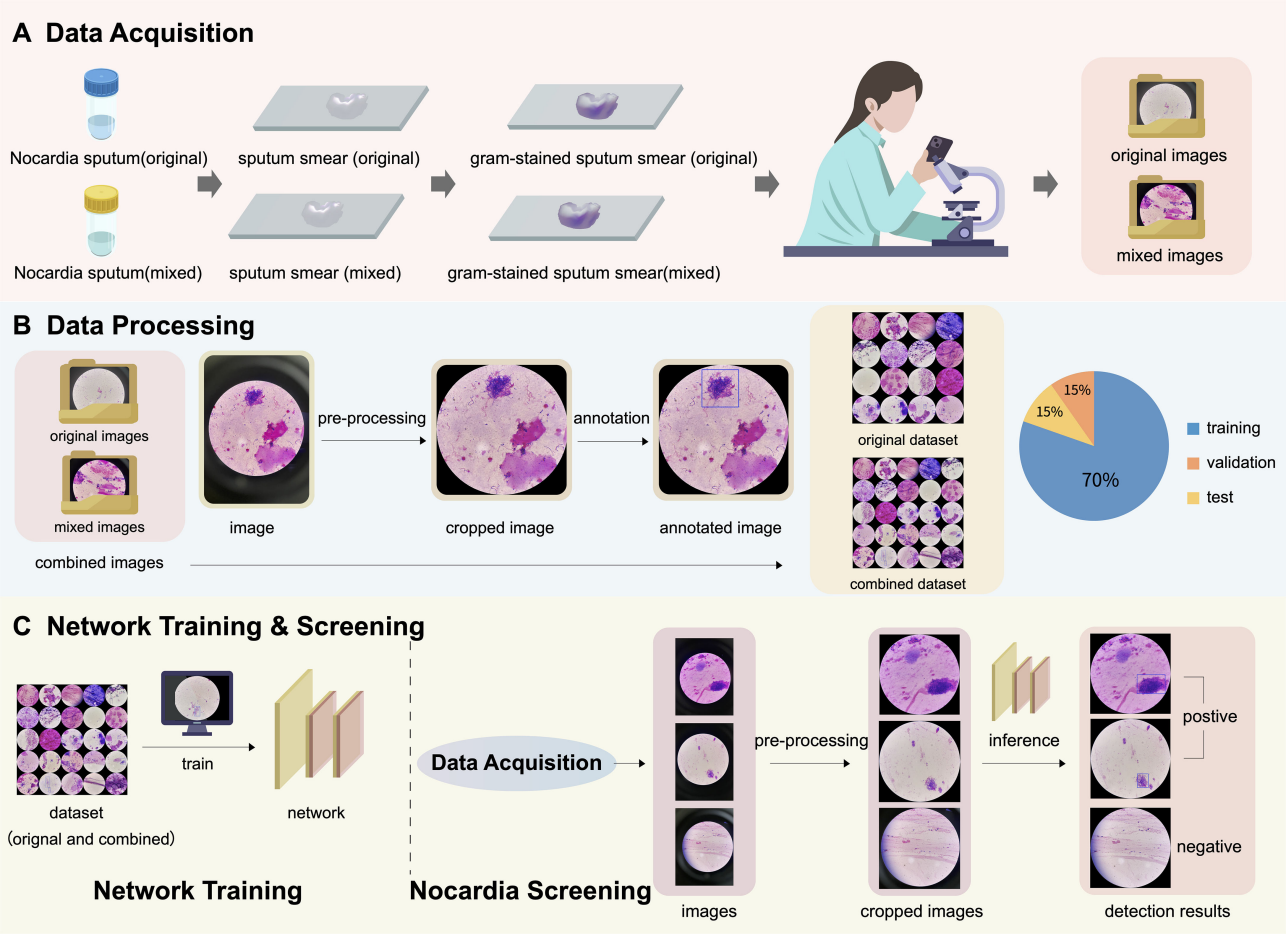
An illustration of the pipeline of the proposed Nocardia screening method, which consists of three steps: **(A)** data acquisition, **(B)** data processing, and **(C)** network training & screening. Note that the combined dataset contains both original and mixed images.

This study was approved by the Ethical Committee of Tongde Hospital of Zhejiang Province with approval number of 2023-077-JY. The whole pipeline of the proposed Nocardia screening method is depicted in **Figure 2**.

### 2.1 Materials

During the period from 2020 to 2023, we collected two Nocardia strains obtained from sputum specimens from two patients. The Nocardia strains were identified as *Nocardia puris* and *Nocardia terpenica* through 16S rRNA sequencing analysis. The sputum smears from the patients were Gram-stained, and then microscopic images of the smears were captured under an OLYMPUS CX23 microscope with a magnification of 1000. The images were captured using the cameras of two smartphones, Apple iPhone 12 and OnePlus 10 Pro, and saved in color mode as JPEG format. All the experiments related to neural networks were conducted on a personal computer equipped with an Intel i7-10700K CPU, 16 GB RAM and an NVIDIA GTX 2070 super GPU with 8 GB VRAM.

#### 2.1.1 Data diversity

In this section, we introduce the methods for enhancing the diversity in both the foreground and background of the images. According to our observation, the diversity of the foreground depends primarily on the morphology of Nocardia, rather than Nocardia strains. Therefore, it is effective to enhance it by increasing the quantity of images. For the background, the Nocardia-positive sputum specimens were mixed with new sputum specimens from patients without Nocardia infection. As a result, a total of 10 mixed sputum specimens were generated, including 2 cases of mucous sputum, 2 cases of saliva sputum, 2 cases of blood sputum, and 4 cases of caseous sputum. With this mixture strategy, many new types of bacteria were incorporated, significantly enhancing the diversity of the image background.

#### 2.1.2 Datasets

A total of 1721 images were captured in our study. Among them, 797 images were identified as Nocardia positive, including 326 originating from the original sputum specimens and 471 from the mixed ones. The remaining 924 images were identified as Nocardia negative, including 766 from the original sputum specimens and 158 from the mixed ones. The composition of these images is also detailed in **Table 1**. These images made up two datasets: the combined dataset containing all 1721 images and the original dataset containing 1092 images captured from the original sputum smears. For each dataset, all the images were randomly divided into three sets: the training set (70%), the validation set (15%), and the test set (15%). The same division configuration was employed for both classification and detection.

**Table 1:** The composition of the datasets.

Subset	original +	original -	mixed +	mixed -	total
**train**	218	520	331	109	1178
**valid**	55	126	75	28	284
**test**	53	120	65	21	259
**total**	326	766	471	158	1721

+, Nocardia positive; -, Nocardia negative.

### 2.2 Data processing

**Figure 3:**
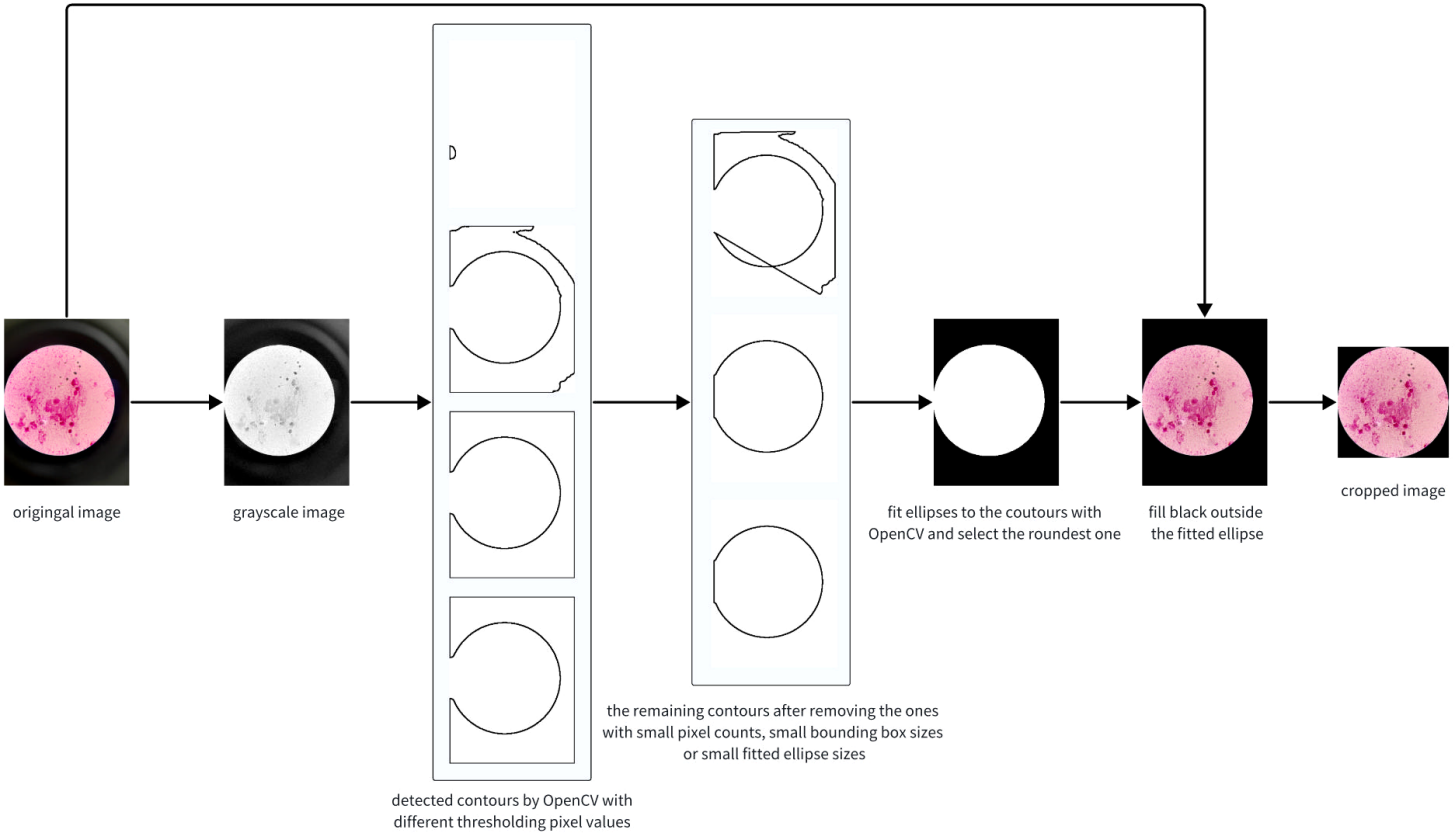
The image cropping pipeline using OpenCV.

As depicted in the cropped image in **Figure 2B**, the pixels outside the microscope field view provide irrelevant information, making it reasonable to crop the image and retain only the content within the field view. It is unwise to crop thousands of images manually; therefore, we propose automatically cropping the images with the OpenCV library (https://docs.opencv.org/3.4/d6/d00/tutorial_py_root.html), as shown in **Figure 3**.

The principal idea of the algorithm is to detect an ellipse for the bright circle and crop the image with its bounding box. Firstly, we convert the image into grayscale. In normal cases, the pixel values of the grayscale image are the weighted average of the RGB values. However, we found that extracting the maximum values in the RGB channels yields better performance. Secondly, we identify contours and fit them to ellipses. Note that contours with few points or small bounding boxes should be dropped. Due to the significant variation in image brightness, using multiple thresholds for contour finding is necessary and crucial for success. Thirdly, the final ellipse is selected based on the largest cropping metric value, where the cropping metric is defined as the ratio of the length of the semi-minor axis to that of the semi-major axis. Finally, we fill the region outside the ellipse with black and crop the original image, preserving only the content inside the bounding box. The algorithm’s pseudo code, written in Python-style, is presented in **Algorithm 1**.

The results showed that more than 99% of the images in the dataset were cropped correctly. After cropping, an average of 40.9% of the pixels were removed, greatly enhancing the ratio of valid pixels, and thereby improving the performance of the networks.

The cropped images were then annotated by three clinical microbiology experts with more than 10 years of experience, using an open-source annotation software named “labelImg” (https://github.com/HumanSignal/labelImg). One of the experts annotated all the sets as the ground truth, while the other two carefully reviewed the annotation results to eliminate potential errors. When performing annotation for detection, a rectangle was manually drawn on the image for each Nocardia instance found in the image, as shown in the annotated image in **Figure 2B**, and the meta-information of the rectangles was stored in text files. After annotation, the detection results could be easily converted to classification annotations. Specifically, an image was classified as positive if it contained at least Nocardia instance; otherwise, it was considered negative.

### 2.3 Network training

#### 2.3.1 Network architecture

**Figure 4:**
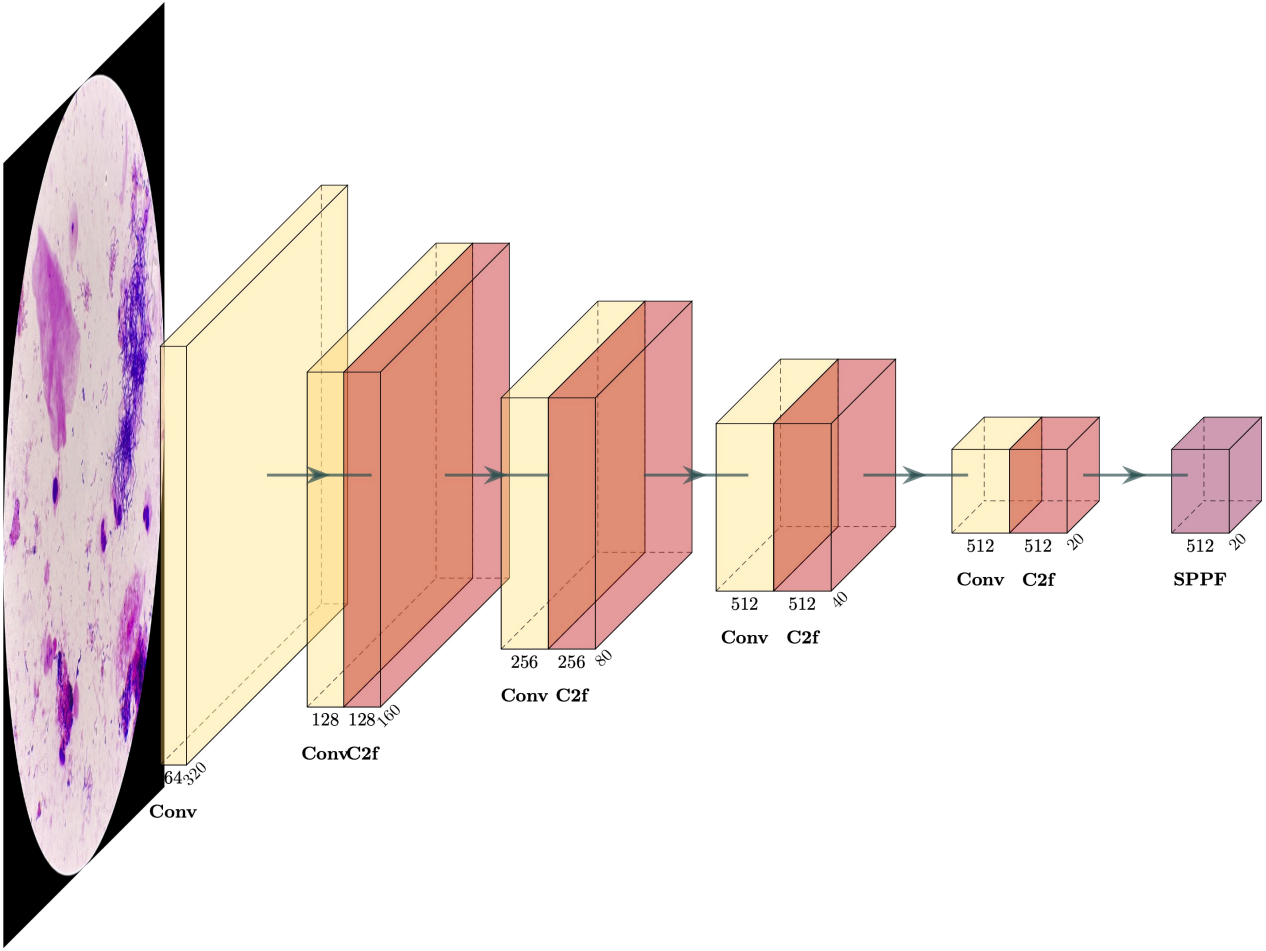
The backbone of the YOLOv8 detection network.

In the proposed Nocardia screening method, the network architecture of YOLOv8 (You Only Look Once version 8) (Redmon et al., 2016; Redmon and Farhadi, 2017; Redmon and Farhadi, 2018; Jiang et al., 2022; Wang et al., 2023a) was adopted for Nocardia detection, namely, marking Nocardia instances with bounding rectangles in the images. Unlike previous detection networks, e.g., R-CNN (Girshick et al., 2014), Fast R-CNN (Girshick, 2015), Faster R-CNN (Ren et al., 2015), and Mask R-CNN (He et al., 2017), that perform multiple predictions for various regions, YOLO performs only one prediction to get all bounding boxes, significantly improving the training and inference efficiency. Meanwhile, it can achieve comparable or even better detection performance than previous methods. The network architecture of YOLOv8 is complicated, and we depict its backbone in **Figure 4**. For more details, we recommend referring to the homepage of YOLOv8 (https://ultralytics.com/yolov8).

**Algorithm 1:** The pseudo code for image cropping using OpenCV

1 function cropImage(image, thresholdList): 2 gray = convertWithMaxValue(image) 3 ellipses = [] 4 for threshold in thresholdList: 5 contours = findContours(gray, threshold) 6 contours = getValidContours(contours) 7 ellipses += [fitEllipse(contour) for contour in contours] 8 bestEllipse = getBestEllipse(ellipses) 9 fillBlackOutOfEllipse(image, bestEllipse) 10 boundingBox = getBoundingBox(bestEllipse) 11 finalImage = cropWithBoundingBox(image, boundingBox) 12 return finalImage

#### 2.3.2 Data augmentation

To improve the performance of the neural network, data augmentation was involved in the pipeline. We applied several different image transformations to the images, including image flipping, rotation, cropping and color changing, which significantly improved the diversity and size of the dataset.

#### 2.3.3 Pre-training

The adopted network can be divided into two functional parts, one for feature extraction, and the other for detection. Researchers found that the feature extraction part has a very strong generalization ability, which can be shared among networks for different tasks, whereas the latter part is to detect specific objects, which should be retrained for each task. Therefore, we started our training process by loading a neural network model which was pre-trained on the large-scale COCO dataset (Lin et al., 2014), which consists of 164k images. This pre-training skill imbues the trained network with powerful feature extraction capabilities.

#### 2.3.4 Training

**Figure 5:**
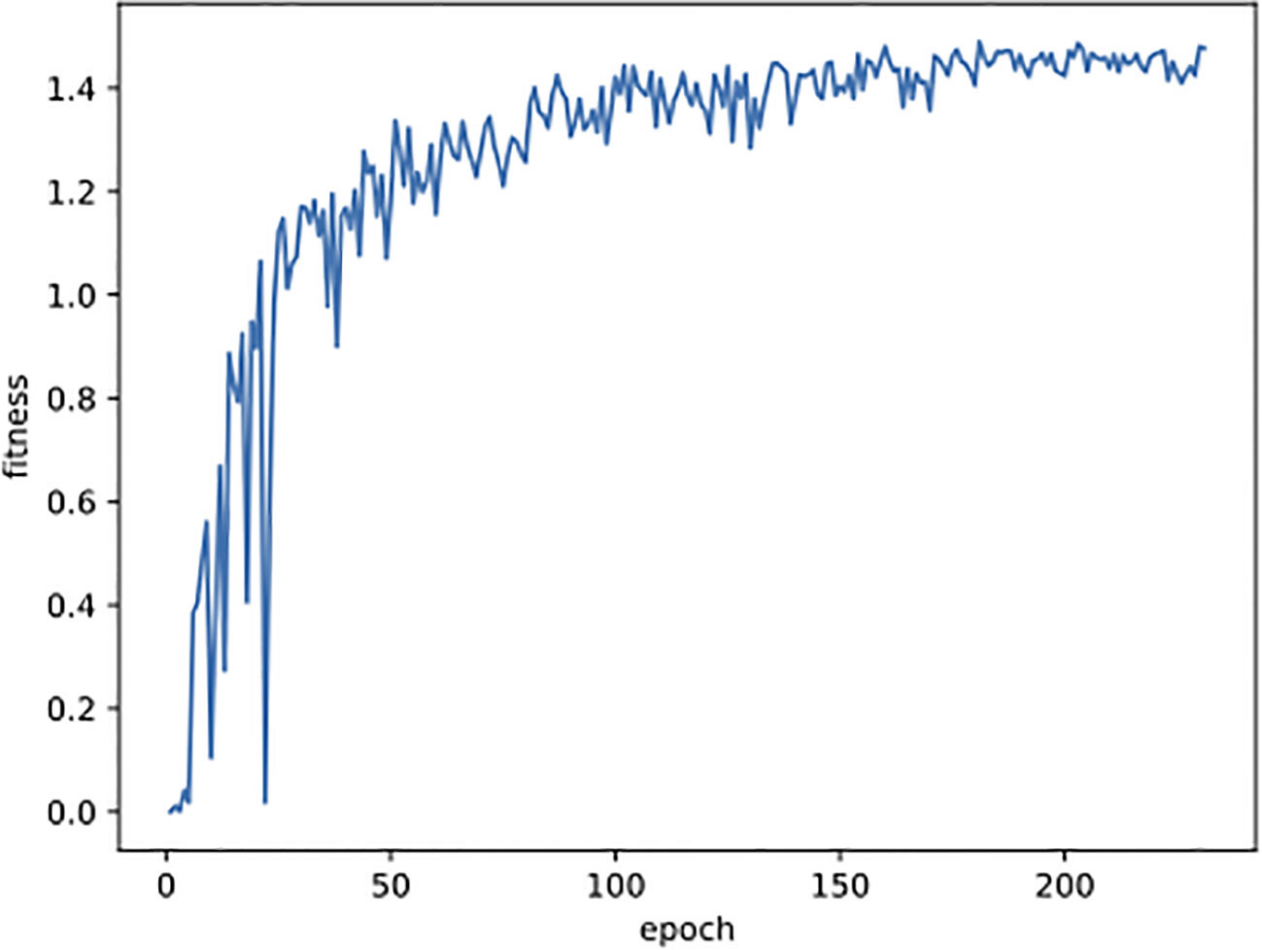
The curve of fitness changing with epoch during the training process on the mixed dataset.


All the images were resized to 640 pixels for both width and height before being used for training, validation, and testing. The YOLO detection network was trained using Stochastic Gradient Descent (SGD) (Bottou, 2010) with a momentum of 0.937 and a batch size of 16. The training process was carried out within 300 epochs, and it would terminate earlier if the fitness didn’t increase for 50 consecutive epochs (for example, see **Figure 5**). The fitness is defined in the following formula, where mAP50 and mAP will be introduced in Section 3.2. Other parameters were all kept the same as YOLOv8 recommended. The training times were 5.6 and 7.0 hours on the original and combined datasets, respectively.

fitness=mAP50×0.1+mAP×0.9

### 2.4 Evaluation

The performances of the trained networks were evaluated on the test sets by comparing the predictions with the ground truth annotation results. The evaluation metrics were accuracy, sensitivity, specificity, positive predictive value (PPV), negative predictive value (NPV), and F-Score, which are calculated with the following formulas:


accuracy=(TP+TN)/(TP+TN+FP+FN)

sensitive=recall=TP/(TP+FN)

specificity=TN/(TN+FP)

PPV=TP/(TP+NP)

NPV=NP/(TN+FN)

precision=TP/(TP+FP)

F−Score=2×precision×recall/(precision+recall)

where TP, TN, FP, and FN are abbreviations for true positive, true negative, false positive, and false negative, respectively.

## 3 Results

### 3.1 Classification

**Figure 6:**
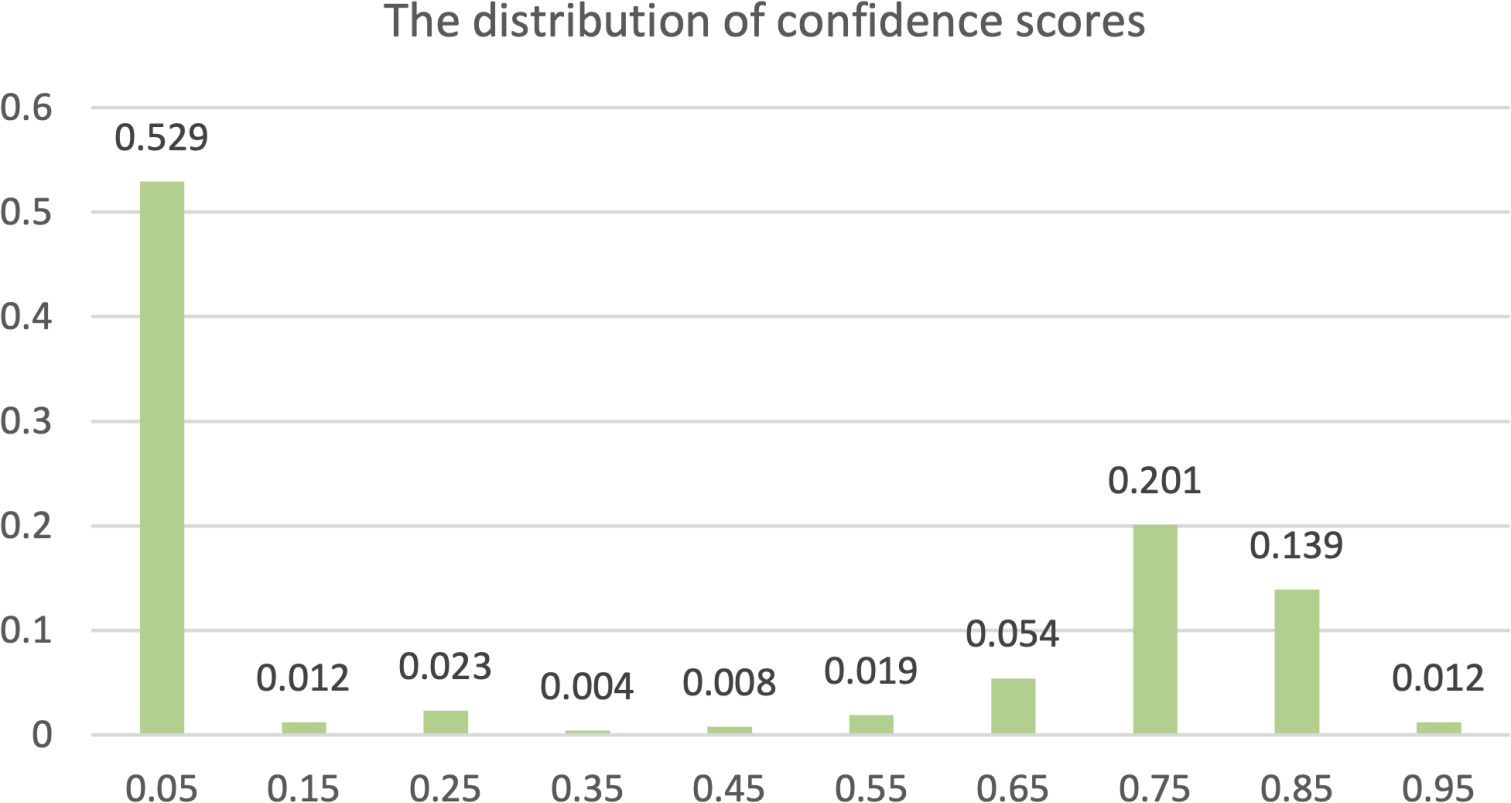
The distribution of confidence scores. Each label x along the horizontal axis represents a range from x-0.05 to x+0.05.

The primary goal of the proposed screening method is to classify whether an image contains Nocardia. For comparison, we conducted experiments with the YOLOv8 detection network (YOLO-det), the YOLOv8 classification network (YOLO-cls), Faster R-CNN, and manual annotation. Note that both YOLO-det and Faster R-CNN are detection networks, but their detection results could be easily converted to classification results. In our experiments, if at least one Nocardia region was detected in an image with a sufficient confidence score, the image would be classified as positive for Nocardia, and vice versa. The distribution of confidence scores is shown in **Figure 6**. Manual annotation was performed by two clinical microbiology experts, and their average metrics were compared with the other methods.

**Figure 7:**
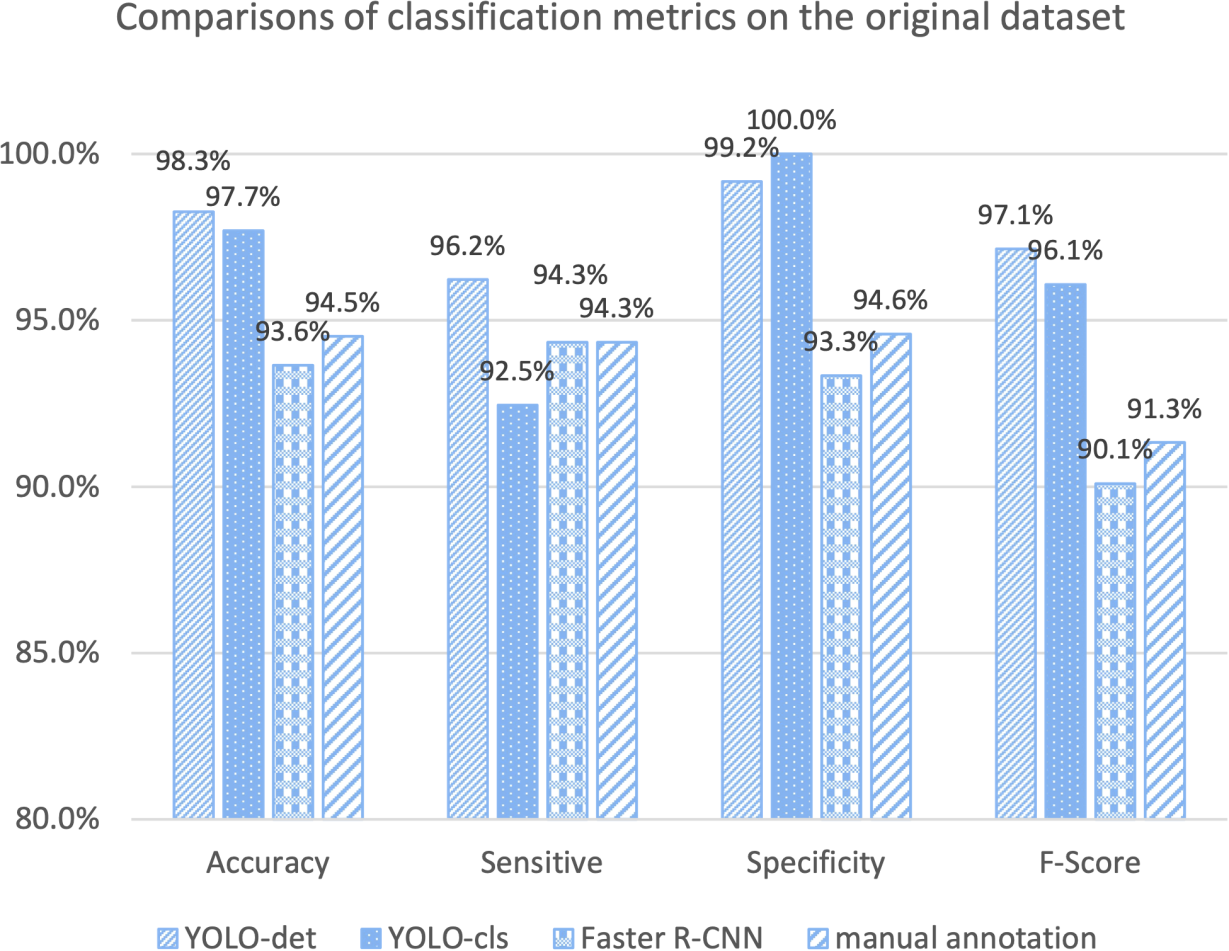
Comparison of classification metrics on the original dataset.

**Figure 8:**
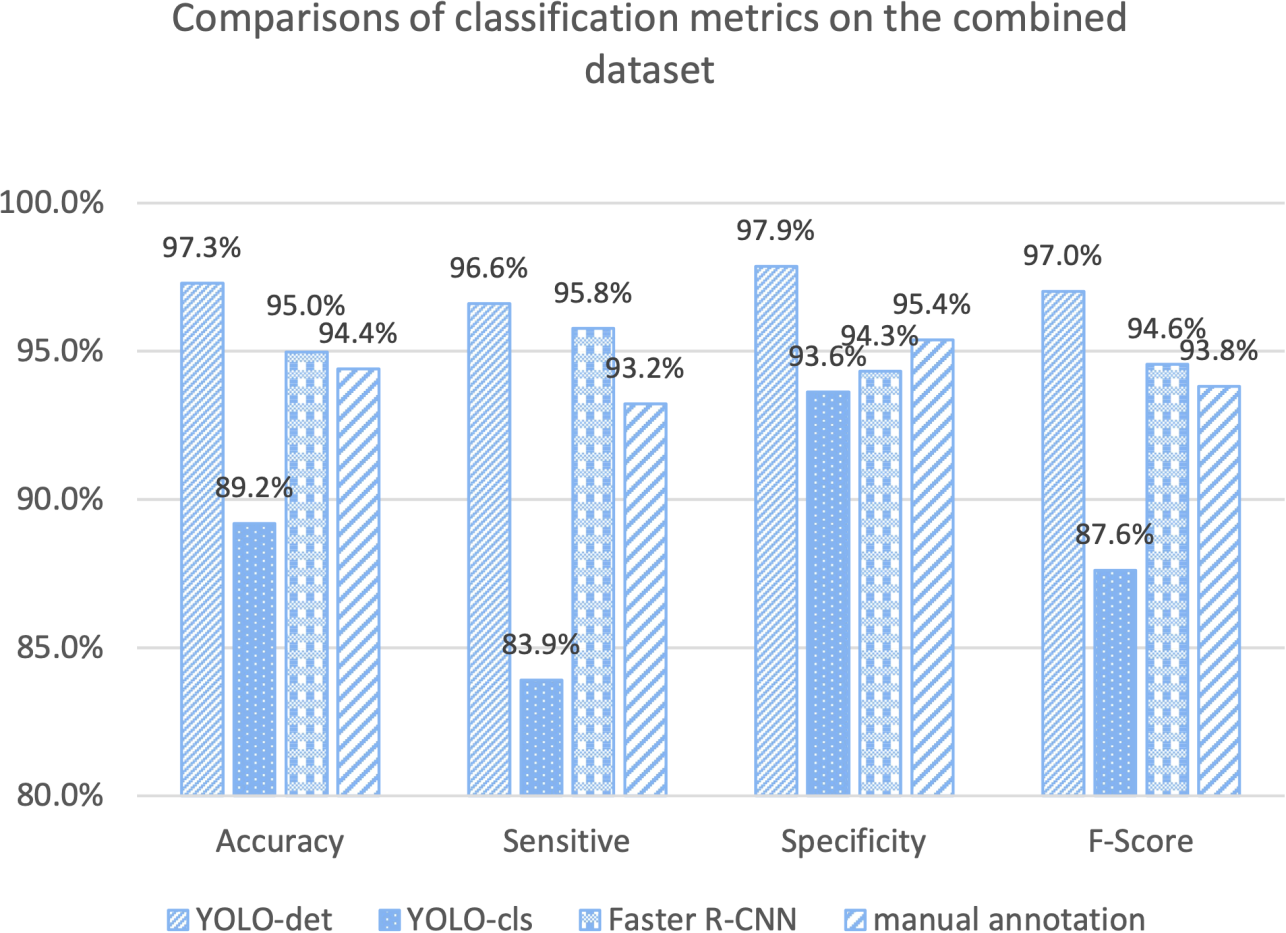
Comparison of classification metrics on the combined dataset.

**Figure 9:**
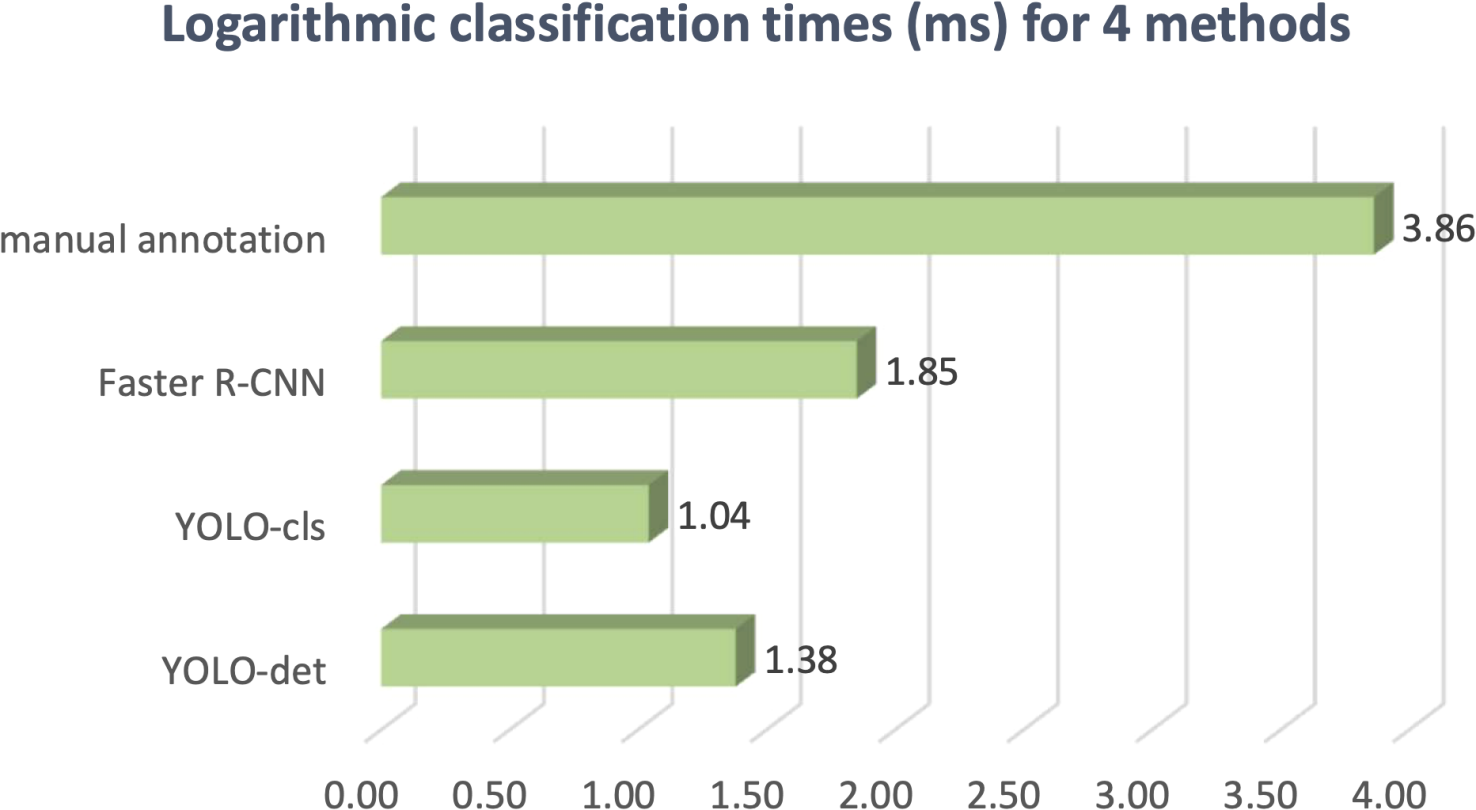
Logarithmic classification times for four methods.

The classification results are compared in **Figures 7** and **8**, and detailed data are recorded in **Table 2**. YOLO-det achieved accuracies of 98.3% and 97.3% on the original and combined datasets, respectively, which were the highest among all the methods on both datasets. The inference times are shown in **Figure 9**, which demonstrates that the classification of YOLO-det was 304 times faster than manual annotation.

**Table 2:** The classification metrics for 4 methods on the original and combined datasets.

Dataset	Method	Accuracy	Sensitive (Recall)	Specificity	PPV	NPV	Precision	F-Score
**original**	**YOLO-det**	**98.3%**	**96.2%**	**99.2%**	**98.1%**	**98.3%**	**98.1%**	**97.1%**
	YOLO-cls	97.7%	92.5%	100.0%	100.0%	96.8%	100.0%	96.1%
	Faster R-CNN	93.6%	94.3%	93.3%	86.2%	97.4%	86.2%	90.1%
	manual annotation	94.5%	94.3%	94.6%	89.1%	97.4%	88.5%	91.3%
**combined**	**YOLO-det**	**97.3%**	**96.6%**	**97.9%**	**97.4%**	**97.2%**	**97.4%**	**97.0%**
	YOLO-cls	89.2%	83.9%	93.6%	91.7%	87.4%	91.7%	87.6%
	Faster R-CNN	95.0%	95.8%	94.3%	93.4%	96.4%	93.4%	94.6%
	manual annotation	94.4%	93.2%	95.4%	94.5%	94.4%	94.4%	93.8%

The bold values show the results of the proposed method (YOLO-det).

### 3.2 Detection

**Figure 10:**
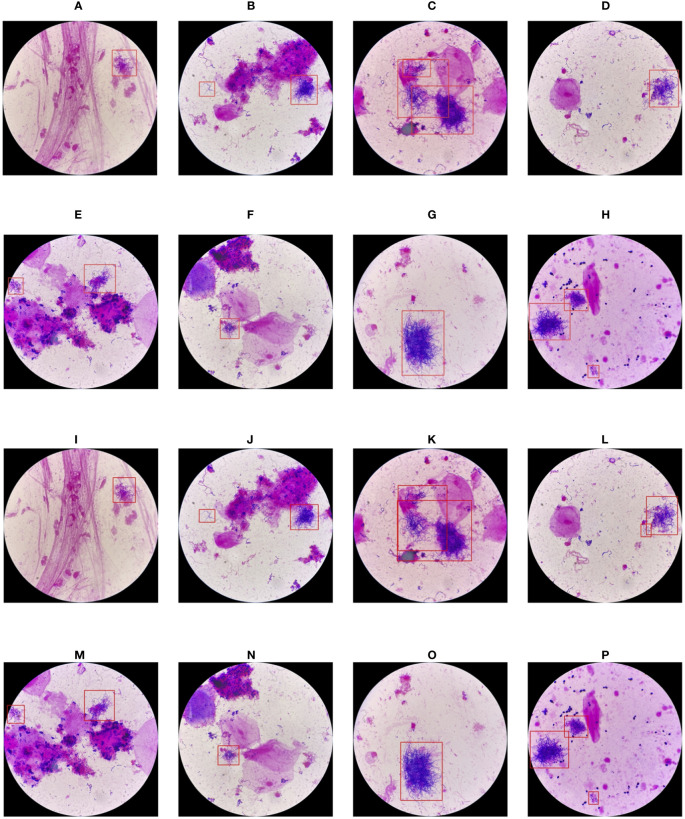
Visualization of the detection results of YOLO-det **(A-H)** and Faster R-CNN **(I-P)**.

The secondary goal of the screening method is to detect Nocardia instances within the images and display the detected locations with bounding boxes, assisting doctors in confirming the presence of Nocardia. The detection results for YOLO-det and Faster R-CNN are visualized in **Figure 10**, and they appear quite similar. To quantify the detection performance, we utilized two metrics: mAP (mean Average Precision) and mAP50 (Lin et al., 2014). These two metrics are both defined based on IoU (intersection over union), which is a common metric measuring the overlap between the predicted bounding box and the ground-truth bounding box. mAP50 corresponds to the precision of matched predictions, where a prediction is considered a match if the IoU is not lower than a threshold of 50%. Similarly, mAP computes the mean prediction with multiple thresholding values ranging from 0.5 to 0.95 with a step size of 0.05. These two metrics measure the quality of detection at different levels, with higher values indicating better detection performance. In **Table 3**, the results show that YOLO-det achieved higher mAP on both datasets, higher mAP50 on the combined dataset, and nearly identical mAP50 on the original dataset, demonstrating superior detection performance over Faster R-CNN.

**Table 3:** The detection metrics for YOLO-det and Faster R-CNN on the original and combined datasets.

Metric	Dataset	YOLO-det	Faster R-CNN
**mAP**	original	**0.425**	0.385
	combined	**0.469**	0.462
**mAP50**	original	**0.780**	0.781
	combined	**0.841**	0.817

The bold values show the results of the proposed method (YOLO-det).

### 3.3 Model generalization

In this section, we assessed the generalization ability of the neural networks under consideration. Each network was trained on the training sets from both the original and combined datasets and subsequently tested on the corresponding test set. As a result, we obtained four different configurations: “o-o”, “o-c”, “c-o”, and “c-c”. Here, “o-c” indicates that the network was trained on the original dataset and tested on the combined dataset, and similar conventions apply to other configurations.

**Figure 11:**
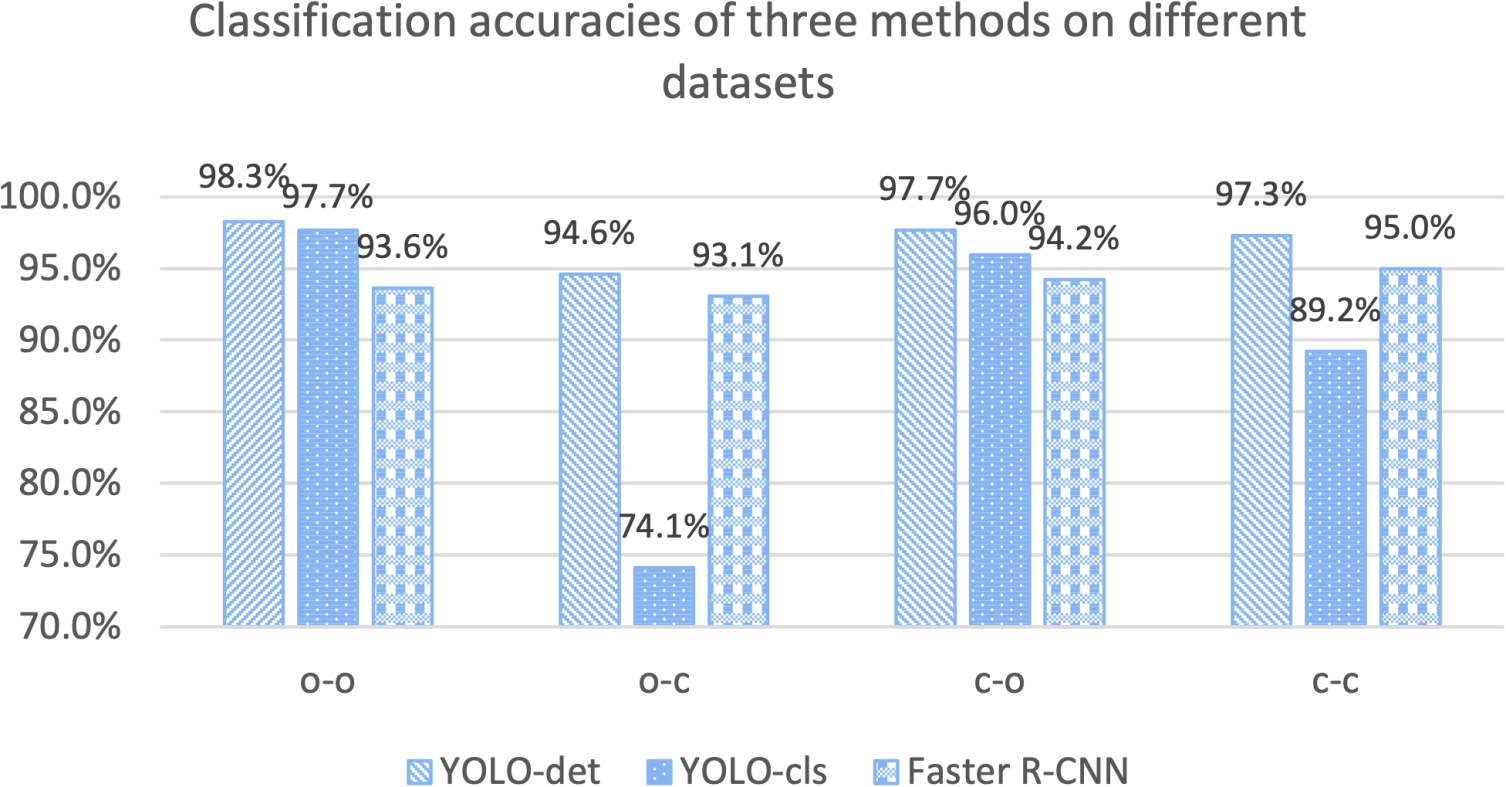
Accuracy comparisons for three methods on different training and testing datasets. “o” stands for the “original dataset”, and “c” stands for the “combined dataset”. The configuration of “o-c” stands for training on the original dataset and testing on the combined dataset. Other configurations are defined similarly.

The classification accuracies for YOLO-det, YOLO-cls, and Faster R-CNN based on these four configurations are illustrated in **Figure 11**. By comparing the accuracies in “o-o” and “o-c”, a substantial accuracy drop is observed for YOLO-cls, whereas the accuracy drops are much slighter for both detection methods. This comparison demonstrates that the detection methods exhibit significantly stronger generalization ability than the classification method.

To validate the generalization ability among different Nocardia strains, we conducted an additional experiment by applying the model trained with two strains directly on a dataset containing a new strain. 74 images were captured from two smears from two patients, including 28 positives and 46 negatives. The Nocardia strains in both smears were identified as Nocardia cyriacigeorgica. Because of the differences in morphology, there was a certain decrease in the confidence scores, so we lowered the thresholding confidence score to 0.1. The results showed that 50% of the positives and 100% of the negatives were correctly classified, yielding an overall accuracy of 81.1%. This result was consistent with experiences in the field of neural networks. Since the model had not encountered instances of the new strain in the training set, it might classify them as negatives, but it would not misclassify negatives as positives. The results of this experiment indicated that the model trained on two strains was able to detect certain instances of a new strain, but with reduced accuracy. Therefore, the model should be trained with more Nocardia strains before being applied in medical practice.

### 3.4 Failure cases

**Figure 12:**
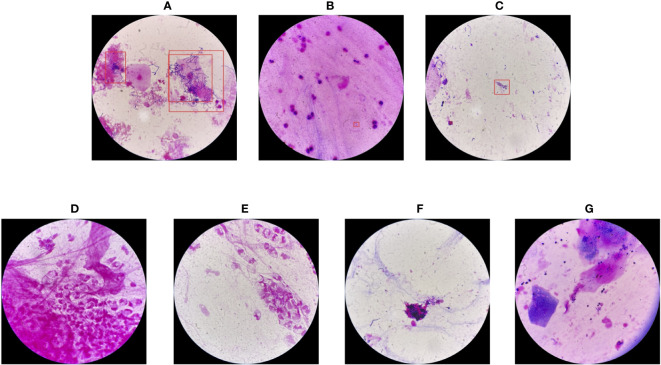
Failure cases of YOLO-det on the combined dataset. The first three cases **(A-C)** are false positives, while the others **(D-G)** are false negatives.

In this section, we present a comprehensive analysis of all seven failure cases corresponding to YOLO-det on the combined dataset, including 3 false positives and 4 false negatives, as illustrated in **Figure 12**.

For the false positives, in image (A), the morphology of the detected bacteria is quite similar to Nocardia, resulting in misclassification. In image (B), the confidence score from the network output was on the boundary between positive and negative, resulting in ambiguous classification. However, image (C) presents a case of clear misclassification. Among the 4 false negatives, the Nocardia instances are challenging to identify because their appearances are difficult to distinguish from the background. As is common in the field of artificial intelligence, accuracy could be further improved by training networks on a larger and more diverse dataset, which we plan to explore in the future.

## 4 Discussion

In this study, we present a novel Nocardia screening method based on the YOLO detection network. To the best of our knowledge, this is the first time neural networks have been applied for Nocardia detection in the field of laboratory testing. The experiments indicated outstanding accuracies of 98.3% and 97.3% on the original and combined datasets, respectively, thereby demonstrating the remarkable effectiveness of the screening method. Notably, the accuracies also surpassed those of manual annotations in the experiments, as illustrated in **Figures 7** and **8**. Beyond the advantage of classification accuracy, the inference time of the network-based method was two magnitudes less than manual annotation, demonstrating the high efficiency of the screening method. Compared to existing laboratory testing methods, such as MALDI-TOF, PCR, and NGS, the proposed network-based method has the advantages of both efficiency and low cost. In conclusion, taking effectiveness, efficiency, and cost-effectiveness into consideration, the neural network-based screening method presents substantial advantages in Nocardia screening over other methods. Its potential to reduce the missed diagnosis rate and improve timeliness can contribute to improving the overall cure rate.

Although most previous works have adopted neural classification networks to determine whether a specific pathogen was present in an image (Zhang et al., 2019; Cai et al., 2020; Kang et al., 2020; Khan et al., 2021; Momeny et al., 2022; Poomrittigul et al., 2022; Trivedi et al., 2023), we propose that it can achieve comparable or even better performance to adopt a detection network, rather than a classification network, in certain scenarios. This assertion is based on three reasons.

1) In the “Classification” section, the results reveal that YOLO-det achieved the highest accuracies among all the methods on both datasets.

2) Beyond accuracy, model generalization ability is a crucial metric. It is well-known that a neural network trained on one dataset may perform poorly on other datasets because of the so called “domain gap” phenomenon. As demonstrated in the “Model Generalization” section, when YOLO-cls was trained on the original dataset but tested on the combined dataset, the accuracy decreased significantly to 74.1%, much lower than those of other configurations. This phenomenon suggests that this network learned specific knowledge from the original dataset, which could not be applied to new images outside the dataset. In contrast, the detection networks exhibited much stronger generalization abilities, making them more practical for Nocardia screening. The enhanced generalization ability could be attributed to their focus on informative parts with different locations and scales, observing a wider range of variances and, consequently, stronger robustness.

3) The detection networks not only determine whether the input image contains Nocardia instances, but also locate them to assist doctors in diagnosis.

Besides YOLO-det, we also tested Faster R-CNN for comparison. In terms of classification accuracy, YOLO-det outperformed Faster R-CNN on both the original and combined datasets. For detection performances, among all the 4 configurations, YOLO-det achieved higher metric values in 3 configurations and nearly identical metric values in the 4th configuration. Overall, YOLO-det showed better results than Faster R-CNN in both classification and detection tasks on our datasets. Nevertheless, one network may not achieve the best performances in all scenarios, and other network architectures (Ren et al., 2015; Liu et al., 2016; Lin et al., 2017; Carion et al., 2020) could also be considered to use, depending on the application scenarios.

Although Nocardia infection is uncommon in patients, we made efforts to capture plenty of images, ensuring sufficient diversity in the morphology of Nocardia instances. Additionally, by mixing the original sputum specimens with new ones from patients without Nocardia infection, the diversity of the background pathogens was significantly enhanced. In **Figure 11**, we can see that the accuracies of the group “c-c” were significantly higher than those of the group of “o-c”, demonstrating the effectiveness of the mixture strategy.

This paper acknowledges several limitations that we plan to address in future research. Firstly, different Nocardia strains exhibit slight variations in morphography. Our neural network model was trained with only two of them, and it did not generalize well to other strains, leading to decreased accuracy. It is recommended to train models on larger datasets that include more strains in order to enhance the models’ generalization ability before applying them in medical practice. Secondly, the quantity of the available Nocardia sputum specimens was limited. Although we alleviated the limitation by capturing plenty of images and introducing a mixture strategy, it is possible to achieve more conclusive results with a larger number of sputum specimens with Nocardia infection. Thirdly, we have not compared YOLO-det with methods other than YOLO-cls, Faster R-CNN and manual annotation. It could be more comprehensive if more neural network architectures were tested for comparison. Lastly, the proposed method should be adopted for screening purposes to reduce missed diagnosis rate, and the results should be further tested with diagnosis techniques before guiding clinicians.

While our study focused on Nocardia screening, the proposed methods, strategies, and conclusions can be extended to other studies. For the screening of pathogens other than Nocardia, neural network-based methods could be applied, due to their demonstrated effectiveness, efficiency, and cost-effectiveness. For a classification task, a detection network could also be considered, which may have higher performance and stronger generalization ability. Additionally, it is effective in improving data diversity by mixing specimens with new ones without the specific pathogens, ultimately enhancing the robustness of the trained networks.

## 5 Conclusion

In this paper, we propose a neural network-based Nocardia screening method. This method adopts the YOLOv8 detection network to identify Nocardia instances in images which are captured from Gram-stained sputum smears under a microscope. The results demonstrates that the proposed method achieves high accuracies of 98.3% and 97.3% on the original and combined datasets, respectively. Our study also reveals that detection networks may outperform classification networks in terms of accuracy and generalization ability in certain scenarios, which could be extended to studies beyond Nocardia screening. Additionally, we also prove that a mixture strategy can effectively enhance data diversity, leading to improved performance of the trained networks.

## Data availability statement

The raw data supporting the conclusions of this article will be made available by the authors, without undue reservation.

## Ethics statement

The studies involving humans were approved by the Ethical Committee of Tongde Hospital of Zhejiang Province. The studies were conducted in accordance with the local legislation and institutional requirements. The ethics committee/institutional review board waived the requirement of written informed consent for participation from the participants or the participants’ legal guardians/next of kin because this study only involves photographs of Gram-stained sputum smears under a microscope. These photographs do not involve patient privacy and cannot be used to identify patient identities.

## Author contributions

HS: Conceptualization, Data curation, Formal Analysis, Investigation, Methodology, Project administration, Resources, Validation, Visualization, Writing – original draft, Writing – review & editing. XX: Conceptualization, Formal Analysis, Investigation, Methodology, Software, Visualization, Writing – original draft, Writing – review & editing. YW: Methodology, Writing – review & editing. JW: Methodology, Writing – review & editing. TD: Data curation, Formal Analysis, Funding acquisition, Methodology, Writing – review & editing.
